# A Time for Global Action: Addressing Girls’ Menstrual Hygiene Management Needs in Schools

**DOI:** 10.1371/journal.pmed.1001962

**Published:** 2016-02-23

**Authors:** Marni Sommer, Bethany A. Caruso, Murat Sahin, Teresa Calderon, Sue Cavill, Therese Mahon, Penelope A. Phillips-Howard

**Affiliations:** 1 Mailman School of Public Health, Columbia University, New York, New York, United States of America; 2 Rollins School of Public Health, Emory University, Atlanta, Georgia, United States of America; 3 UNICEF Headquarters, New York, New York, United States of America; 4 UNICEF Bolivia, La Paz, Bolivia; 5 WaterAid, London, United Kingdom; 6 Liverpool School of Tropical Medicine, Liverpool, United Kingdom

## Abstract

Marni Sommer and colleagues reflect on priorities needed to guide global, national, and local action to address girls' menstrual hygiene management needs in schools.

**Summary Points**

- There is an absence of guidance, facilities, and materials for schoolgirls to manage their menstruation in low- and middle-income countries (LMICs).
- Formative evidence has raised awareness that poor menstrual hygiene management (MHM) contributes to inequity, increasing exposure to transactional sex to obtain sanitary items, with some evidence of an effect on school indicators and with repercussions for sexual, reproductive, and general health throughout the life course.
- Despite increasing evidence and interest in taking action to improve school conditions for girls, there has not been a systematic mapping of MHM priorities or coordination of relevant sectors and disciplines to catalyze change, with a need to develop country-level expertise.
- Columbia University and the United Nations Children's Fund (UNICEF) convened members of academia, nongovernmental organizations, the UN, donor agencies, the private sector, and social entrepreneurial groups in October 2014 (“MHM in Ten*”*) to identify key public health issues requiring prioritization, coordination, and investment by 2024.
- Five key priorities were identified to guide global, national, and local action.

## Introduction

A lack of adequate guidance, facilities, and materials for girls to manage their menstruation in school is a neglected public health, social, and educational issue that requires prioritization, coordination, and investment [1]. There are growing efforts from academia, the development sector, and beyond to understand and address the challenges facing menstruating schoolgirls in low- and middle-income countries (LMIC) [1]. A body of research has documented menstruating girls’ experiences of shame, fear, and confusion across numerous country contexts and the challenges girls face attempting to manage their menstruation with insufficient information, a lack of social support, ongoing social and hygiene taboos, and a shortage of suitable water, sanitation and waste disposal facilities in school environments [2–7]. The accruing evidence reveals the gender discriminatory nature of many school environments, with female students and teachers unable to manage their menstruation with safety, dignity, and privacy, negatively impacting their abilities to succeed and thrive within the school environment [7–9]. Poor school attainment reduces girls’ economic potential over the life course, impacts population health outcomes [9–12], and also extends to girls’ sexual and reproductive health outcomes, self-esteem, and sense of agency [4,7,8].

Despite increasing evidence about the challenges girls face managing menstruation in school in LMIC countries and growing efforts to address these challenges, there has not been a concentrated effort at global or national levels to identify key priorities to catalyze action to transform the school-going experiences of girls. The “MHM in Ten” initiative was organized by Columbia University and the United Nations Children's Fund (UNICEF) in New York City in October 2014 to systematically map out a ten-year agenda for overcoming the menstrual hygiene management (MHM)-related barriers facing schoolgirls.

Significant recent events supported the rationale for organizing such a meeting, illustratively including intense discussion around the inclusion of MHM in the post-2015 sustainable development goals, the investment by the Canadian government (Global Affairs Canada) to support MHM research and programming in 14 countries, the annual cohosting of the MHM in Water, Sanitation and Hygiene (WASH) in Schools virtual conference by UNICEF and Columbia University, designation of May 28 as “Menstrual Hygiene Day,” and the development of a new puberty policy for the education sector with a focus on menstruation education and MHM by the United Nations Educational, Scientific and Cultural Organization (UNESCO). This paper briefly describes the state of the evidence on MHM in schools, the remaining knowledge gaps, and potential action for making progress on the ten-year agenda.

### Current Evidence: Knowledge and Gaps

In 2014, there were over 250 million girls aged 10–14 years of age living in less-developed countries, and nearly 56 million living in least-developed countries [13]. Although reliable evidence on the average age of menarche in many countries is lacking [14], the vast majority of girls will experience their first menstruation during this age range.

Growing evidence suggests the gendered impacts of inadequate WASH facilities in LMIC schools influence the participation of girls [15,16]. Much of the MHM research, conducted across sub-Saharan Africa, Asia, and South America, has concentrated on understanding girls’ experiences of the onset of menstruation and the subsequent WASH challenges they face managing their menstruation in school [1]. Girls have indicated receiving inadequate guidance prior to their first menstrual period and experiencing fear, shame, and embarrassment managing menstruation, particularly while in school [4,6,7,17,18]. Studies have shown girls lack water, soap, privacy, and space to change [19]; adequate time to manage their menses comfortably, safely, and with dignity [20–22]; and hygienic sanitary products and sometimes underwear [7,23,24]. The latter lack may increase girls’ vulnerability to coercive sex and subsequent sexual and reproductive health harms to obtain money to buy sanitary products [25,26]. Female schoolteachers in many contexts also struggle to manage their menstruation comfortably and privately in schools and may be hard to retain in the absence of adequate WASH facilities; less evidence and action exist in relation to female teachers [27], but such work is needed. Many school systems have a predominance of male administrators and teachers, who may be unaware of or reluctant to talk about the challenges that schoolgirls and female teachers are facing [28]. Further contributing to unsupportive social environments at school, boy students report having little understanding about menstruation, and some tease and bully girls because they do not understand girls’ behaviors during menstruation [28–30]. This evidence has provided insights for some nascent programming and policy actions generally focused on three key MHM elements in school: the provision of MHM guidance, fostering an enabling physical and social school environment, and the distribution of menstrual products [2,31].

However, there remains a paucity of empirical evidence quantifying the extent and intensity of girls’ challenges managing menstruation, with few studies examining causal associations and little experimental evidence available to demonstrate the effectiveness of MHM interventions for health and schooling [32]. There has also been insufficient research examining the impact of inadequate MHM guidance or environments on schoolgirls’ levels of self-esteem, their self-efficacy to manage their menstruation in school, and their ability to concentrate in class when menstruating in schools that lack adequate WASH facilities or sensitized teachers and peers. There is also insufficient research examining the impact of interventions aimed at reducing menstrual-related bullying and improving girls’ self-confidence. This lack of evidence makes it difficult to promote recommendations to national governments, nongovernmental organizations, and others interested in integrating MHM into education and health strategies, and it reduces global buy-in to move this agenda forward. Lastly, while policy makers have called for increased measurement of school attendance, dropout, and educational attainment in relation to MHM, demonstrating quantitative associations with school absence in limited studies to date has shown minimal effect [5,33,34], despite strong qualitative evidence from girls’ narratives [4,7,17,26,35]. Similarly, self-reporting of reproductive tract infections among adolescent schoolgirls has also been shown to be unreliable without laboratory confirmation [36].

There are currently two distinct arguments put forward in relation to generating attention and resources to address inadequate MHM in schools. One frames the issues in relation to meeting the basic human rights and dignity of girls (and female teachers), while the second focuses on how ongoing barriers to effective MHM may contribute to negative health and education outcomes for girls. The global community has to some degree achieved consensus on the importance of the first but now needs to focus increased resources on generating adequate evidence for action on the second.

### A Need for Collaboration across Sectors: MHM in Ten Aimed at Catalyzing Discussions across Sectors

Both the human rights argument and the need to improve MHM for health and educational reasons provide strong rationales for engagement from multiple sectors. However, while the MHM challenges facing pubescent girls in LMIC require cross-sectoral responses, funding streams and structures are needed to support sustainable activities by institutions and government ministries. Convergence between departments to prevent duplication and gaps similarly requires attention [37].

To date, much of the leadership and activities on MHM in schools has been through the WASH sector. The education sector has been less engaged, even though girls’ school experiences are negatively impacted if they are distracted, uncomfortable, or unable to participate because of anxiety over menstrual leakage and odor [7] or without the support of teachers, adequate latrines [20], or a place to rest if menstrual cramps become painful [4,31]. Girls’ sexual and reproductive health underscores the importance of engagement from the education sector given the evidence showing that educated girls are more likely to delay first sex, have fewer sexual partners, and use contraception and are less likely to become infected with HIV/AIDS. In terms of other population health gains, they are also more likely to have their children vaccinated and attend school and have healthier families [9,38–41]. MHM has yet to be included within the numerous activities underway to improve girls’ educational outcomes in LMICs.

There exists a window of opportunity to reach girls at menarche, as their bodies are biologically changing and they are encountering profound new social dynamics within their families and communities [42,43]. Many girls in LMIC receive no or factually incorrect guidance prior to menarche about the normal physiological process of menstruation or the pragmatics of MHM [8]. This in turn results in numerous misconceptions about their own fertility, creating vulnerability to adolescent pregnancy if girls are sexually active [7,9]. The adolescent sexual and reproductive health (SRH) sector is called on to expand its focus and intervention timing beyond contraception (i.e., family planning) and disease prevention to include puberty and menstrual care guidance.

A range of stakeholders (see S1 Table) [44] discussed school environments, educational outcomes, SRH, gender, social beliefs, menstrual management products, and political commitment at the local, national, and global levels. The group included an array of expertise, comprising academics with varied experience conducting qualitative studies as well as randomized trials, nongovernmental organizations (NGOs) working in both advocacy and policy roles, bilateral donors and foundations, small and global level private sector corporations, and a range of UN agency perspectives. The under-representation of LMIC government representatives (i.e., Ministries of Education), country program managers, engineers, youth voices, and other key stakeholders at this first initiative was identified, and the organizers made a commitment to address this in subsequent meetings.

### Priorities for Action

MHM in Ten participants defined a joint aim for the ten-year agenda: “Girls in 2024 around the world are knowledgeable about and comfortable with their menstruation and able to manage their menses in school in a comfortable, safe, and dignified way.”

Five key priorities were identified to achieve this vision by 2024 (Table 1). The priorities are not intended to be sequential as some may happen in parallel:

1. **Build a strong cross-sectoral evidence base for MHM in schools for prioritization of policies, resource allocation, and programming at scale.** Specifically, rigorous impact evaluations of the most essential, cost-effective, and efficient interventions to implement in schools are needed, as well as a broader array of appropriate measures to capture the health and educational impacts of inadequate MHM. National-level research is still required to assure that policies, resources, and programs are appropriate and effective.
2. **Develop and disseminate global guidelines for MHM in schools with minimum standards, indicators, and illustrative strategies for adaptation, adoption, and implementation at national and subnational levels.** The absence of accepted global guidelines and indicators for what to implement in schools and how to monitor interventions is paralyzing governments, school systems, and other practitioners who want guidance for action.
3. **Advance MHM in schools activities through a comprehensive evidence-based advocacy platform that generates policies, funding, and action across sectors and at all levels of government.** There is a need for improved advocacy around MHM given taboos in many countries that hinder open discussion about addressing MHM in schools and the stakeholder engagement needed at all levels (i.e., governments, donors, parents teachers, and students).
4. **Allocate responsibility to designated government entities for the provision of MHM in schools (including adequate budget and monitoring and evaluation [M&E]) and the reporting to global channels and constituents,** recognizing that scalable impact on MHM in schools will only occur when national governments take responsibility for and perceive MHM as a priority for education systems, which highlights their role for change in the coming ten years.
5. **Integrate MHM and the capacity and resources to deliver inclusive MHM into the education system. The education sector recognizes and demonstrates MHM as an integral part of its resources, plans, budgets, services, and performance monitoring and delivers inclusive educational service to all children and adolescents.** Numerous components of action are needed within a given educational system to assure action and monitoring of MHM interventions in schools, and such actions need to be inclusive of vulnerable groups, including girls with disabilities.

**Table 1:** Illustrative cross-sectoral actions to meet priorities.

Stakeholder Group	Role
Governments	Work with their own constituents to “break the silence” on menstruation within their respective institutions and populations
	Allocate resources to implement structural changes in schools
UN agencies	Provide technical support on the development of policies, guidelines, and standards for improving MHM in schools
Researchers	Work with donors, governments, and NGOs to fill the gap in empirical evidence on the relationship between poor MHM and lost schooling, attainment, dropout, self-esteem, self-efficacy, sexual and reproductive health harms, and girls’ inequity
	Provide research evidence on the utility and cost-effectiveness of interventions, operational research on implementation strategies, and support of the policy agenda
Nongovernmental organizations	Work with governments, donors, the private sector, and communities to implement sustainable programming that reaches girls at all income levels
	Collaborate with researchers to rigorously monitor and evaluate programs
Private sector and social entrepreneurs	Use extensive marketing and product distribution skills and entrepreneurial approaches to build momentum for social change
	Construct messages that provide accurate and nondiscriminatory information to girls
Donors	Support research that aims to quantify the extent and severity of girls’ MHM-related challenges and the impact of programs created to ameliorate them

### Recognized Challenges and Opportunities to Achieving Goals

There are challenges to moving forward the MHM agenda in the next ten years; however, there is reason for optimism.

There are competing priorities in the health and education spheres for the existing development resources for adolescent girls. Integrating MHM into existing programming and policy could be a noncompetitive and cost-effective approach. The provision of puberty and menstruation education booklets to girls getting the human papillomavirus (HPV) vaccine has been suggested as a potential integrated strategy.

The majority of existing efforts aimed at addressing MHM have emerged from the WASH community, yet the WASH sector alone cannot advance the MHM agenda in schools. MHM in schools must be supported by a diverse range of actors globally and within countries (i.e., Ministries of Education, Health, Finance, Sanitation and Water, and Women and Children’s Affairs), including any other government specific entities at the country level holding responsibility due to the legal and competence frame of each country (see Table 1 for examples). The current 14-country WASH in Schools (WinS) for Girls program led by UNICEF with support from the Canadian government aims to conduct local research and use findings to generate a platform for action. To ensure multisector involvement, the program has required participating countries form in-country working groups with representatives from the government, local research institutions, and NGOs so varied feedback can be integrated throughout the process. The lessons learned from this effort can serve as a model for multiactor efforts moving forward.

Evidence-based interventions require robust research to support cost-effective programming, and the latter will require an increase in dedicated resources to support the trials needed to generate the data for decision making. Funding is now occurring; for example, the UK-based Department for International Development, Medical Research Council, and Wellcome Trust are supporting a large-scale trial evaluating the effect of menstrual cups on Kenyan girls’ school and SRH outcomes.

### The Way Forward

Participants identified key next steps to reach these priorities:

- A first step is for stakeholders to develop an operational strategy for the MHM in Ten agenda that includes a M&E component for assessing current status and progress in addressing the five priorities. This includes collection of standardized indicators generated at the national level. Buy-in from national governments and schoolgirls themselves is essential.
- A second step is to build collaboration and strengthen research capacity across countries and regions of the world on MHM. It is critical to identify existing, or foster new, MHM experts and actors in each country, whether through strengthening research capacity of in-country academics to conduct local research on MHM or through efforts to mobilize MHM stakeholders within the country to generate collaboration and activity. A global repository for existing evidence, programs, and policies on MHM in schools will provide a platform for decision making. Fostering essential research would be supported through a research consortia. A research concept note detailing the existing gaps in the evidence has been developed [45]; however, a collation and review of the lessons learned to date from existing programming and policy is needed.
- A third step is to coordinate progress and share outcomes, bringing together country- and global-level stakeholders. A steering group comprising global and local expertise, meeting at least annually, and supported through a UN agency would facilitate this.

## Conclusion

There have been numerous early accomplishments in the nascent MHM field in the last few years. In order to reach the vision of girls around the world being knowledgeable about and comfortable with their menstruation and able to manage it safely and with dignity in school, global support of the priorities identified at MHM in Ten is required to make sustainable change by 2024.

## Supporting Information

S1 Table Organizations participating in the first MHM at Ten Meeting. (DOCX)

## Abbreviations

HPV: human papillomavirus
LMIC: low- and middle-income country
M&E: monitoring and evaluation
MHM: menstrual hygiene management
NGO: nongovernmental organization
SRH: sexual and reproductive health
UNESCO: United Nations Educational, Scientific and Cultural Organization
UNICEF: United Nations Children's Fund
WASH: water, sanitation and hygiene
WinS: WASH in Schools
