# Supplementary material for: A Time for Global Action: Addressing Girls’ Menstrual Hygiene Management Needs in Schools
Source: PLoS Med. 2016 Feb 23;13(2):e1001962. doi: 10.1371/journal.pmed.1001962 (PMC4764363; doi:10.1371/journal.pmed.1001962)
Supplement: S1 Table — (DOCX) [file pmed.1001962.s001.docx]

**Table S1 Organizations participating in the first MHM at Ten Meeting**

| Institution | Expertise (all MHM) |
| --- | --- |
| Columbia University | Social science, MHM and WASH education in schools, gender studies, and policy |
| LMIC representatives | In-country field researchers |
| Bill and Melinda Gates Foundation | Sexual and reproductive health, funding |
| Emory University | Epidemiology, public health, MHM and WASH education in schools |
| FHI360 | WASH in schools, education |
| International Rescue Committee | Refugee health and policy |
| Liverpool School of Tropical Medicine / Kenya Medical Research Institute | Epidemiology, systematic review, trials, adolescent sexual and reproductive health |
| London School of Hygiene and Tropical Medicine | WASH, reproductive health, clinical, systematic reviews, research |
| McGill University | Social science, sexual health, gender, international education |
| National Institute of Health Science | Social science, public health |
| Proctor & Gamble | Marketing, local enterprise |
| Save the Children Fund | Support country-based research and implementation programs |
| Sesame Street | Media and communication |
| Sustainable Health Enterprises (SHE) | Local enterprise |
| United Nations Educational, Scientific and Cultural Organization (UNESCO) | Education, policy |
| United Nations Girls’ Education Initiative (UNGEI) | Education, policy |
| United Nations Children's Fund (UNICEF) | Global and national support for implementation of WASH and MHM in schools; global and country level representation |
| University of Kwa Zulu Natal | Research |
| USAID/OFDA | Refugees, funding research and implementation |
| WASH Advocates | Advocacy, WASH, environmental and gender studies |
| Water Supply and Sanitation Collaborative Council (WSSCC) | Advocacy, WASH, environmental, hygiene implementation |
| WaterAid | International and national WASH program support; MHM guidelines |
| World Health Organization (WHO) | Adolescent sexual and reproductive health, policy, program implementation |
| Women Deliver | Advocacy, communications, gender |
| World Bank Group | Promotion of WASH, funding research and program development |
